# Supplementary material for: The pupil responds spontaneously to perceived numerosity
Source: Nat Commun. 2021 Oct 12;12:5944. doi: 10.1038/s41467-021-26261-4 (PMC8511033; doi:10.1038/s41467-021-26261-4)
Supplement: Supplementary file 1 — Supplementary Information [file 41467_2021_26261_MOESM1_ESM.pdf]

Supplementary Information to “The pupil responds spontaneously to perceived numerosity”

Elisa Castaldi[1,2], Antonella Pomè[2], Guido Marco Cicchini[3], David Burr[2,4,*], Paola Binda[1]

[1] Department of Translational Research and New technologies in Medicine and Surgery, University of Pisa, Pisa, Italy

[2] Department of Neuroscience, Psychology, Pharmacology and Child Health, University of Florence, Florence, Italy.

[3] Institute of Neuroscience, National Research Council, Pisa, Italy

[4] School of Psychology, University of Sydney, Camperdown, NSW, Australia

* Corresponding Author: Prof. David Burr

E-mail: davidcharles.burr@unifi.it

Supplementary Notes

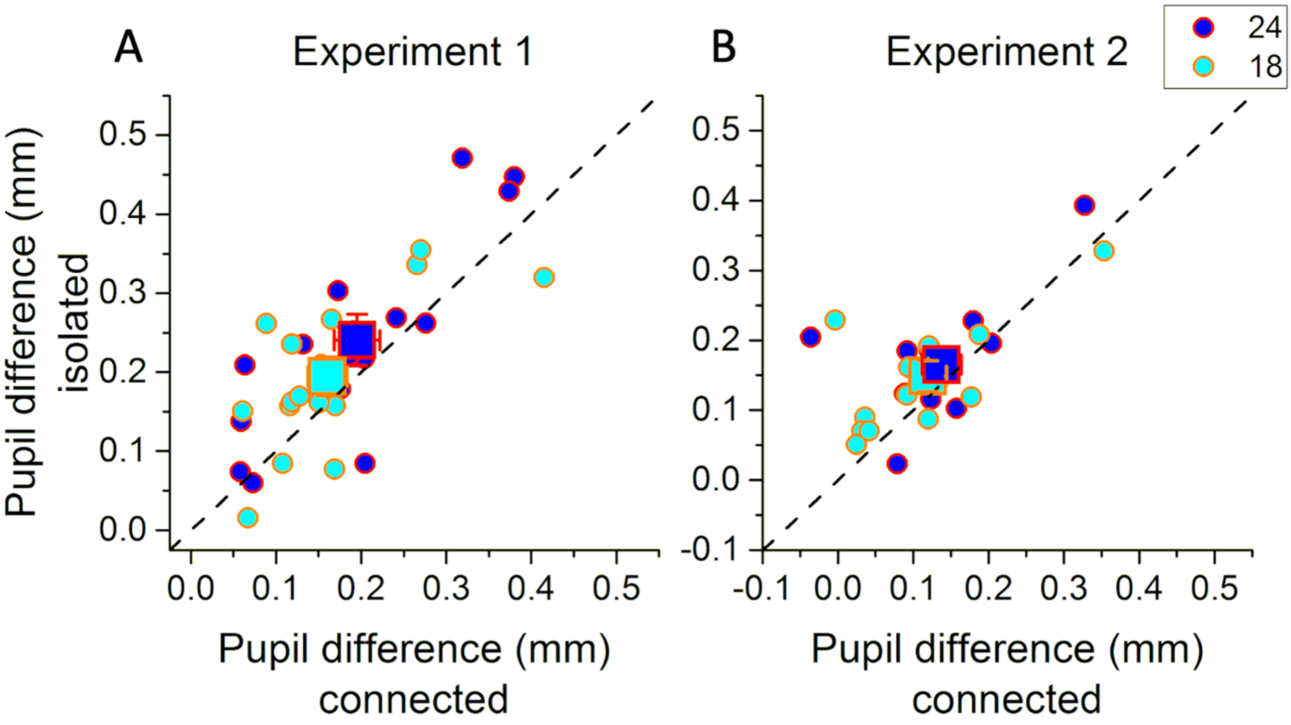

Supplementary Figure 1. Individual participants' pupil difference values, averaged in the interval 1-6 secs after stimulus onset for experiment 1 (A, N = 16 participants) and experiment 2 (B, N= 13 participants). Results from the isolated dots condition are plotted against those for the connected dots condition (different colors for the two numerosities, see legend). Colored circles are results for individual participants; squares show averages across participants, with error bars representing 1 SEM. The results from the first experiment (A) were replicated in the second one (B). Source data are provided as a Source Data file.

Supplementary Note 1: Eye movement analysis

To verify fixation stability, we measured the variability of eye position around its mean (fixation point) on each trial. Specifically, the dispersion of eye position was calculated as the bivariate confidence ellipse area (BCEA)[1,2] , that is the area of a bivariate contour ellipse encompassing eye position samples, defined as:

$$BCEA = 2 * k * \sigma_H * \ \sigma_V * (1 - \rho)^{0.5} \qquad (1)$$

Where k is the confidence limit for the ellipse, σ_H and σ_V are the standard deviation of eye positions in the horizontal and vertical meridian respectively, and ρ is the product-moment

correlation of these two position components. As in previous studies[1,2], we set k=1.14, thus the probability of a given observation falling within the ellipse was 68% ($1-e^{-k}$).

To test whether differences in eye-movement patterns could explain the results, for each participant and condition we averaged the BCEA values across trials and stimulus colors and entered these values in the same 2x2 repeated measure ANOVA used for pupil analyses. No main effect or interaction was significant, suggesting that eye-movements or unstable fixation cannot explain the pupil difference between conditions (Supplementary Figure 2 A, main effect of connectedness: $F(1,15) = 0.002$, $p = 0.96$, log10BF = –0.6; main effect of numerosity: $F(1,15) = 0.4$, $p = 0.55$, log10BF = –0.4; connectedness by numerosity interaction: $F(1,15) = 3.6$, $p = 0.078$, log10BF = –0.4). Similarly, eye-movements cannot explain the pupil difference between conditions in the experiment 2 (Supplementary Figure 2 B, main effect of connectedness: $F(1,12) = 1.4$, $p = 0.25$, log10BF = –0.5; main effect of numerosity: $F(1,12) = 0.8$, $p = 0.38$, log10BF = –0.3; interaction between connectedness and numerosity: $F(1,12) = 1.7$, $p = 0.22$, log10BF = –0.2).

Supplementary Note 2: Perceived brightness

To test whether apparent brightness could explain the pupil difference across conditions, the perceived relative brightness was evaluated by a forced-choice procedure where 10 participants judged which of two sequentially presented stimuli (all white-dot stimuli with displaced-lines for the isolated dots: Figure 1A) appeared brighter. One was the standard, always 18-dot isolated, the other the test, being one of the four conditions (18 or 24 dots, isolated or connected). To make the task more realistic, and give participants confidence in their matching, the standard varied over five levels of luminance, from 206 to 229 cd/m^2, while the tests all had the intermediate luminance levels (218 cd/m^2). Supplementary Figure 2 C shows the proportion of times the test was judged brighter than the standard, only for the condition where the physical luminances were matched.

As in all the analyses, the strongest expected effects are between the extreme conditions, 18 connected and 24 isolated dots. The proportion brighter was slightly higher for the 24 isolated dot condition, but the difference did not approach significance ($t(9) = 0.7$, $p = 0.52$ LogBF = –0.4). Assuming that the errors in brightness are normally distributed, the difference of these two conditions corresponds to 0.48 JNDs, where a JND is defined as the difference in brightness necessary to change the proportion brighter from 0.5 to 0.74.

No terms of the 2x2 ANOVA for all four matches reached significance (main effect of connectedness $F(1,9) = 1.7$, $p = 0.23$, log10BF = –0.1; main effect of numerosity $F(1,9) = 0.2$, $p = 0.64$, log10BF = –0.5; connectedness by numerosity interaction $F(1,9) = 1.1$, $p = 0.32$, log10BF = –0.3).

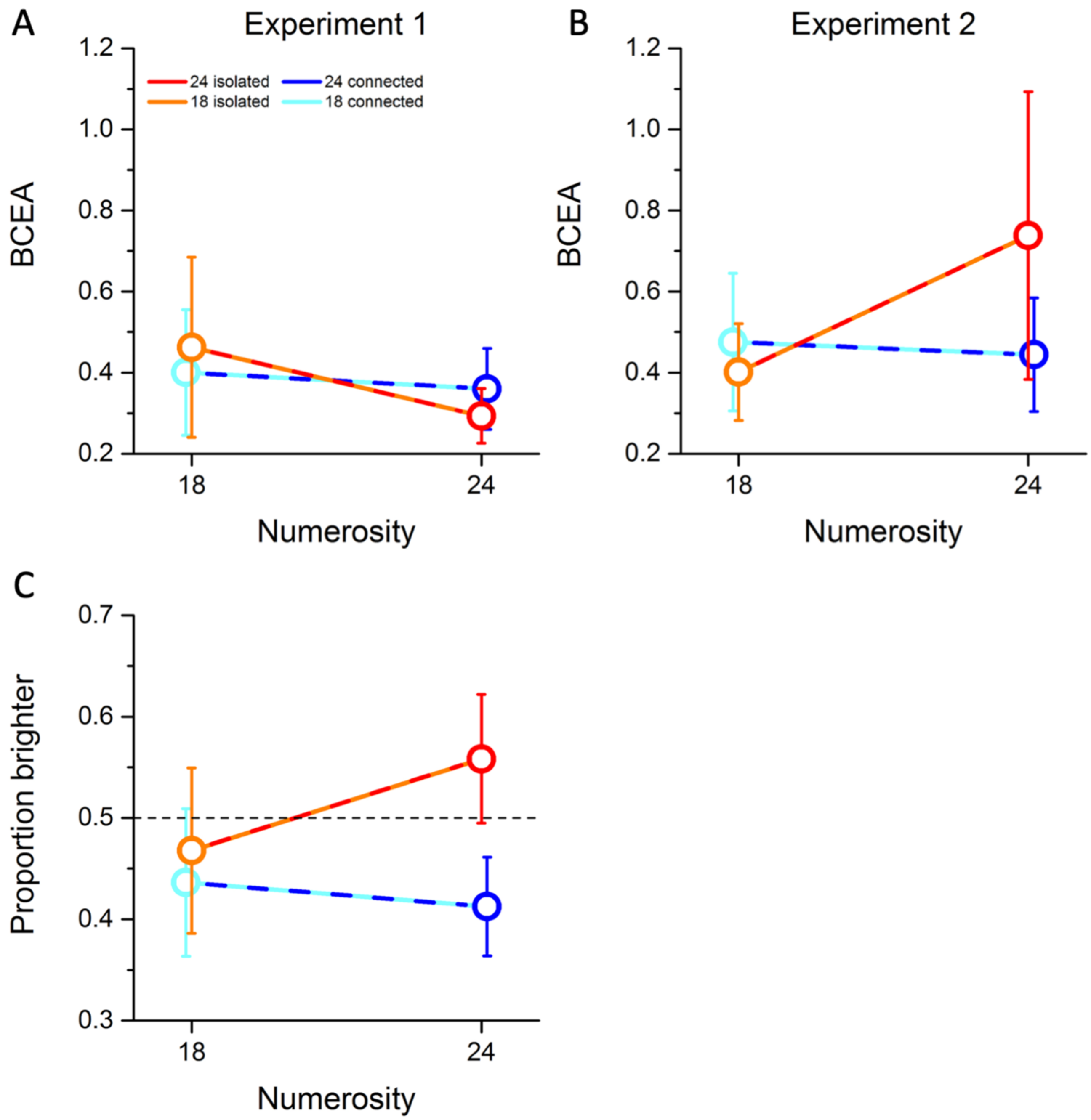

Supplementary Figure 2. Control analyses on eye movements and brightness (color-code in legend). (A) Gaze deviations quantified by the average bivariate confidence interval area (BCEA) for the four conditions tested in experiment 1 (displaced-lines condition, N = 16 participants). These were computed for each participant by averaging across color (black and white), then averaged across participants. The format is the

same as for pupil difference in main Figure 3C&F, yet the pattern is clearly different, implying that eye-movements cannot explain the pupil difference between conditions. Error bars are 1 SEM. (B) Same as A, for experiment 2 (removed-lines condition, N = 13 participants). The results from the first experiment (A) were replicated in the second one (B). (C) Data from brightness matching experiment (N = 10 participants), reporting the average proportion of trials when stimuli of the four conditions were judged to be brighter than standard (18 isolated dots) of matched luminance. Note that the orange symbol refers to matches between identical stimuli. Error bars are SEM. Repeated measures ANOVAs were performed to test for BCEA and brightness perception differences between numerosity and connectedness levels. Significance of main effects and interactions are reported in text. The brightness matching experiment was performed once. Source data are provided as a Source Data file.

Supplementary Tables

		n	τ	δ	Time to peak	R^2
Experiment 1	Pupil difference	2.7(0.2)	452.5(65.7)	164.8(14.1)	755.9(42.7)	0.88(0.02)
	Black stimuli	2.3(0.1)	549.4(54.1)	122.2(20.6)	789.8(55.9)	0.85(0.04)
	White stimuli	2.7(0.2)	463.1(52.8)	184.2(12.0)	826.6(48.6)	0.83(0.02)
		n	τ	δ	Time to peak	R^2
Experiment 2	Pupil difference	2.3(0.1)	533.8(63.3)	147.5(20.7)	790.41(70.3)	0.78(0.06)
	Black stimuli	2.5(0.1)	531.5(67.3)	107.4(25.4)	843.3(85.1)	0.85(0.03)
	White stimuli	3.0(0.4)	413.08(46.8)	170.4(15.3)	892.5(88.4)	0.76(0.06)

Supplementary Table 1. Parameters of the pupil response function, with format mean (SEM) for the two experiments separately. The table reports the pupil response function parameters (n, τ, δ and time to peak) for each participant and for the pupil difference, black and white stimuli, and goodness of fit (R^2) averaged across connectedness and numerosity.

Beta- weights		18 dots connected	18 dots isolated	24 dots connected	24 dots isolated
Experiment 1	Sustained predictor				
	Pupil difference	0.80(0.15)	0.97(0.17)	0.95(0.18)	1.16(0.19)
	Black stimuli	0.48(0.09)	0.58(0.09)	0.47(0.11)	0.62(0.12)
	White stimuli	-0.13(0.07)	-0.16(0.07)	-0.28(0.07)	-0.33(0.07)
	Transient on predictor				
	Pupil difference	0.15(0.03)	0.18(0.03)	0.19(0.03)	0.19(0.03)
	Black stimuli	-0.03(0.02)	-0.01(0.02)	-0.01(0.02)	-0.01(0.02)
	White stimuli	-0.18(0.03)	-0.19(0.04)	-0.20(0.03)	-0.21(0.03)

Transient off predictor				
Pupil difference	-0.10(0.02)	-0.10(0.01)	-0.12(0.02)	-0.11(0.02)
Black stimuli	-0.13(0.02)	-0.13(0.02)	-0.12(0.02)	-0.11 (0.01)
White stimuli	-0.03(0.02)	-0.03(0.01)	-0.01(0.01)	0.00 (0.02)

Supplementary Table 2. Beta values estimated by the fitting procedure of experiment 1. The table reports the beta values for each predictor estimated by fitting the pupil difference and pupil change time courses for each condition (with format mean (SEM)).

Beta- weights		18 dots connected	18 dots isolated	24 dots connected	24 dots isolated
Experiment 2	Sustained predictor				
	Pupil difference	0.37(0.10)	0.54(0.08	0.50(0.08)	0.61(0.08)
	Black stimuli	0.27(0.11)	0.45(0.09)	0.42(0.10)	0.50(0.09)
	White stimuli	-0.05(0.12)	-0.04(0.12)	0.01(0.12)	-0.11(0.10)
	Transient on predictor				
	Pupil difference	0.15(0.03)	0.15(0.03)	0.14(0.03)	0.16(0.03)
	Black stimuli	0.02(0.02)	0.02(0.03)	0.01(0.03)	0.03(0.03)
	White stimuli	-0.14(0.03)	-0.13(0.03)	-0.13(0.02)	-0.13(0.02)
	Transient off predictor				
	Pupil difference	-0.10(0.03)	-0.07(0.02)	-0.10(0.03)	-0.09(0.02)
	Black stimuli	-0.13(0.02)	-0.11(0.02)	-0.11(0.02)	-0.12(0.02)
	White stimuli	-0.02(0.01)	-0.04(0.01)	-0.01(0.01)	-0.03(0.02)

Supplementary Table 3. Same as Supplementary Table 2, but reporting beta values for experiment 2.

Supplementary References

1. Raveendran, R. N., Babu, R. J., Hess, R. F. & Bobier, W. R. Transient improvements in fixational stability in strabismic amblyopes following bifoveal fixation and reduced interocular suppression. *Ophthalmic Physiol Opt* **34**, 214–225 (2014).
2. Binda, P. & Lunghi, C. Short-Term Monocular Deprivation Enhances Physiological Pupillary Oscillations. *Neural Plasticity* **2017**, 1–10 (2017).
