# Supplementary material for: The pupil responds spontaneously to perceived numerosity
Source: Nat Commun. 2021 Oct 12;12:5944. doi: 10.1038/s41467-021-26261-4 (PMC8511033; doi:10.1038/s41467-021-26261-4)
Supplement: Supplementary file 3 — Reporting Summary [file 41467_2021_26261_MOESM3_ESM.pdf]

nature research

Corresponding author(s): Prof. David C. Burr
Last updated by author(s): Aug 9, 2021

Reporting Summary

Nature Research wishes to improve the reproducibility of the work that we publish. This form provides structure for consistency and transparency in reporting. For further information on Nature Research policies, see our Editorial Policies and the Editorial Policy Checklist.

Statistics

For all statistical analyses, confirm that the following items are present in the figure legend, table legend, main text, or Methods section.

n/a	Confirmed	
☐	☒	The exact sample size (*n*) for each experimental group/condition, given as a discrete number and unit of measurement
☐	☒	A statement on whether measurements were taken from distinct samples or whether the same sample was measured repeatedly
☐	☒	The statistical test(s) used AND whether they are one- or two-sided<br>*Only common tests should be described solely by name; describe more complex techniques in the Methods section.*
☐	☒	A description of all covariates tested
☐	☒	A description of any assumptions or corrections, such as tests of normality and adjustment for multiple comparisons
☐	☒	A full description of the statistical parameters including central tendency (e.g. means) or other basic estimates (e.g. regression coefficient) AND variation (e.g. standard deviation) or associated estimates of uncertainty (e.g. confidence intervals)
☐	☒	For null hypothesis testing, the test statistic (e.g. *F*, *t*, *r*) with confidence intervals, effect sizes, degrees of freedom and *P* value noted<br>*Give P values as exact values whenever suitable.*
☐	☒	For Bayesian analysis, information on the choice of priors and Markov chain Monte Carlo settings
☒	☐	For hierarchical and complex designs, identification of the appropriate level for tests and full reporting of outcomes
☐	☒	Estimates of effect sizes (e.g. Cohen's *d*, Pearson's *r*), indicating how they were calculated

Our web collection on statistics for biologists contains articles on many of the points above.

Software and code

Policy information about availability of computer code

Data collection	Stimuli were generated and presented using custom codes and Psychtoolbox-3 routines under Matlab R2019a. Data collection was performed using custom codes, the Psychtoolbox extension for Eyelink and built-in functions from the EyeLink 1000 system (SR research) under Matlab R2019a.
Data analysis	Statistical tests were conducted with Jasp 0.14.1 or with custom codes running under Matlab.

For manuscripts utilizing custom algorithms or software that are central to the research but not yet described in published literature, software must be made available to editors and reviewers. We strongly encourage code deposition in a community repository (e.g. GitHub). See the Nature Research guidelines for submitting code & software for further information.

Data

Policy information about availability of data

All manuscripts must include a data availability statement. This statement should provide the following information, where applicable:
- Accession codes, unique identifiers, or web links for publicly available datasets
- A list of figures that have associated raw data
- A description of any restrictions on data availability

Data are available at the following doi47: 10.5281/zenodo.5168707;
Target URL: https://doi.org/10.5281/zenodo.5168707.

Field-specific reporting

Please select the one below that is the best fit for your research. If you are not sure, read the appropriate sections before making your selection.

☐ Life sciences ☒ Behavioural & social sciences ☐ Ecological, evolutionary & environmental sciences

For a reference copy of the document with all sections, see nature.com/documents/nr-reporting-summary-flat.pdf

Behavioural & social sciences study design

All studies must disclose on these points even when the disclosure is negative.

Study description	Quantitative data have been collected for the current study. The study relies on a within-subjects design.
Research sample	Participants were university students of psychology, biology and engeneering. Sixteen participants (six males, mean age: 30 years old) with normal or corrected to normal vision participated in the study. All participants took part to the main pupillometry experiment, and fourteen of these participated in the numerosity discrimination experiment. Thirteen participants took part to a second pupillometry and psychophysical experiment. The sample is representative and sample size was based on a previus study measuring the same contruct (numerosity perception) with a different eyetracking parameter (saccades).<br><br>Castaldi, E., Burr, D., Turi, M. & Binda, P. Fast saccadic eye-movements in humans suggest that numerosity perception is automatic and direct. Proc. R. Soc. B. 287, 20201884 (2020).
Sampling strategy	Sample size was based on a previus study measuring the same contruct (numerosity perception) with a different eyetracking parameter (saccades).<br><br>Castaldi, E., Burr, D., Turi, M. & Binda, P. Fast saccadic eye-movements in humans suggest that numerosity perception is automatic and direct. Proc. R. Soc. B. 287, 20201884 (2020).<br><br>A random sampling procedure was used.
Data collection	Measurements were made in a quite dark room with participants sitting in an experimental booth surrounded by thick black curtains so that the only light source was the stimulus display (LCD monitor screen, 1280 x 720 pixels, refresh rate 60 Hz). Participants sat at 57 cm from the screen, with head stabilized by chin rest. Pupil diameter was monitored at 500 Hz with an EyeLink 1000 system (SR research) with infrared camera mounted below the screen, recording from the left eye. Before each session, eye position was linearized by a standard 9-points calibration routine.<br>The experimenter was in the testing room and initiated the experiment without interfering with data collection. Experimental conditions were varied pseudorandomly and they were not under the experiementer's control. The researcher was blinded to experimental condition.
Timing	Data collection started in October 2020 and ended in January 2021.
Data exclusions	Data from all participants were included in the analysis. No data were excluded from the analyses.
Non-participation	No participants dropped out/declined partecipation.
Randomization	This is a study in a single group of subjects. All experimental manipulations were within subject.

Reporting for specific materials, systems and methods

We require information from authors about some types of materials, experimental systems and methods used in many studies. Here, indicate whether each material, system or method listed is relevant to your study. If you are not sure if a list item applies to your research, read the appropriate section before selecting a response.

Materials & experimental systems

n/a	Involved in the study
☒	☐ Antibodies
☒	☐ Eukaryotic cell lines
☒	☐ Palaeontology and archaeology
☒	☐ Animals and other organisms
☐	☒ Human research participants
☒	☐ Clinical data
☒	☐ Dual use research of concern

Methods

n/a	Involved in the study
☒	☐ ChIP-seq
☒	☐ Flow cytometry
☒	☐ MRI-based neuroimaging

Human research participants

Policy information about studies involving human research participants

Population characteristics	See above
Recruitment	Participants were recruited through advertisement among univeristy students. No selection biases are expected.
Ethics oversight	The research was approved by the local ethics committee (Commissione per l'Etica della Ricerca, University of Florence, n. 111 on the 7 July 2020) and was in accordance with the Declaration of Helsinki. All participants gave written informed consent prior to the study.

Note that full information on the approval of the study protocol must also be provided in the manuscript.
